# Identifying and assessing a prognostic model based on disulfidptosis-related genes: implications for immune microenvironment and tumor biology in lung adenocarcinoma

**DOI:** 10.3389/fimmu.2024.1371831

**Published:** 2024-05-22

**Authors:** Jin Wang, Kaifan Liu, Jiawen Li, Hailong Zhang, Xian Gong, Xiangrong Song, Meidan Wei, Yaoyu Hu, Jianxiang Li

**Affiliations:** School of Public Health, Suzhou Medical College of Soochow University, Jiangsu, Suzhou, China

**Keywords:** disulfidptosis, LASSO, prognostic model, lung cancer, immune infiltration

## Abstract

**Introduction:**

Lung cancer, with the highest global mortality rate among cancers, presents a grim prognosis, often diagnosed at an advanced stage in nearly 70% of cases. Recent research has unveiled a novel mechanism of cell death termed disulfidptosis, which is facilitated by glucose scarcity and the protein SLC7A11.

**Methods:**

Utilizing the least absolute shrinkage and selection operator (LASSO) regression analysis combined with Cox regression analysis, we constructed a prognostic model focusing on disulfidptosis-related genes. Nomograms, correlation analyses, and enrichment analyses were employed to assess the significance of this model. Among the genes incorporated into the model, CHRNA5 was selected for further investigation regarding its role in LUAD cells. Biological functions of CHRNA5 were assessed using EdU, transwell, and CCK-8 assays.

**Results:**

The efficacy of the model was validated through internal testing and an external validation set, with further evaluation of its robustness and clinical applicability using a nomogram. Subsequent correlation analyses revealed associations between the risk score and infiltration of various cancer types, as well as oncogene expression. Enrichment analysis also identified associations between the risk score and pivotal biological processes and KEGG pathways. Our findings underscore the significant impact of CHRNA5 on LUAD cell proliferation, migration, and disulfidptosis.

**Conclusion:**

This study successfully developed and validated a robust prognostic model centered on disulfidptosis-related genes, providing a foundation for predicting prognosis in LUAD patients.

## 1 Introduction

Lung cancer remains the leading cause of cancer-related death worldwide, and its mortality accounts for approximately 18% of all types of cancer (1). Non–small cell lung cancer (NSCLC) is the most common lung cancer subtype, and it comprises two major histological types: lung squamous cell carcinoma (LUSC) and lung adenocarcinoma (LUAD). In addition to conventional therapies such as surgery, chemotherapy and radiotherapy, targeted therapy and immunotherapy for lung cancer have also developed rapidly in recent years. However, these therapies can only benefit some patients and have many limitations, such as side effects and high costs (2, 3). Nearly 70% of patients with NSCLC are initially diagnosed at a locally advanced stage and suffer from a poor prognosis (4). The 5-year survival rate is less than 3% for patients with advanced NSCLC (5). Therefore, exploring new diagnostic and prognostic markers is an important way to improve the early diagnosis and prognosis of lung cancer.

In the past few years, an increasing number of forms of cell death have been discovered, providing more possibilities for humans to combat various diseases (6). Activating specific forms of death through agonist treatment can provide new strategies for cancer treatment. Recent research has revealed a novel form of cell death, disulfidptosis, which is a form of cell death induced by glucose deficiency and SLC7A11 (7, 8). Specifically, disulfidptosis was triggered when cells with high SLC7A11 protein expression were subjected to glucose starvation. Treatment with glucose transporter (GLUT) inhibitors can induce disulfidptosis in cancer cells with high SLC7A11 expression without significant toxicity to normal tissues, thus effectively inhibiting tumor growth (7). This new form of death opens new doors for the development of cancer treatment strategies. Although the basic concept of disulfidptosis has been proposed, its detailed mechanisms remain unclear, especially its role across different cell types and under disease conditions. Currently, GLUT inhibitors are the only known inducers of disulfidptosis, highlighting the limited understanding of their mechanisms and therapeutic targets (9).

**Figure 1:**
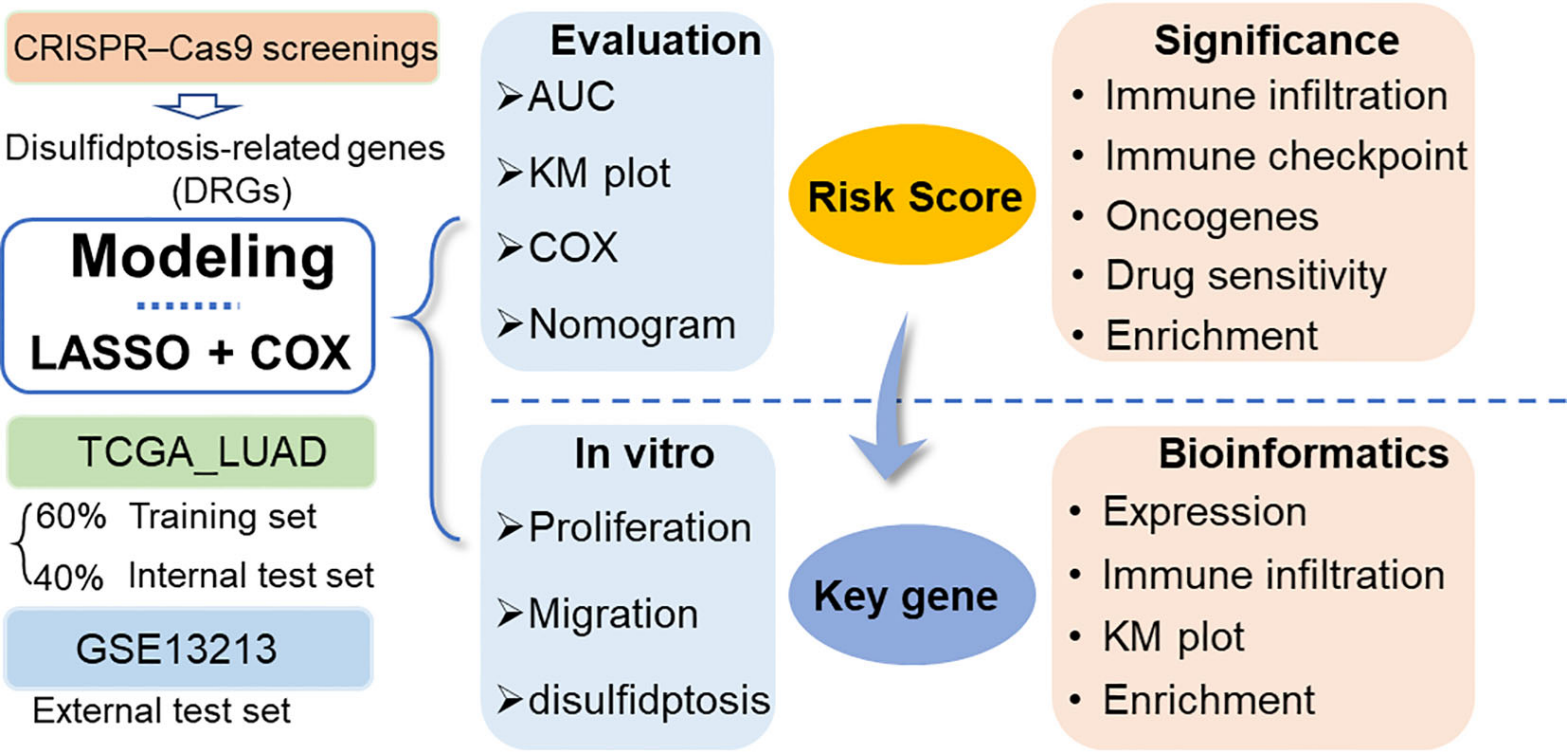
Workflow diagram of this study. DRGs: disulfidptosis-related genes. LASSO, least absolute contact and selection operator. AUC, area under the curve; TCGA, cancer genome map; LUAD, lung adenocarcinoma.

In this study, disulfidptosis-related genes (DRGs) identified from CRISPR–Cas9 screenings were obtained from a previous study (8), and they were used to establish a prognostic model based on the LUAD dataset in the TCGA database and another LUAD dataset in the GEO database using the least absolute contact and selection operator (LASSO) and Cox regression analysis (**Figure 1**). The model-derived risk factors were further analyzed for associations with immune cell infiltration, tumor suppressor gene expression, tumor-related biological functions and drug sensitivity. Moreover, the key genes in the model were further validated by *in vitro* assays.

## 2 Methods

### 2.1 Data collection

The LUAD data of 572 patients, including 59 normal tissues adjacent to cancer tissues, 513 tumor tissues and corresponding clinical information, were retrieved from The Cancer Genome Map (TCGA) database. The expression profile and clinical results are open and accessible. To validate the prognostic model based on the TCGA LUAD cohort, another LUAD dataset (GSE13213) was retrieved from the Gene Expression Omnibus (GEO) database as an external validation dataset. The GSE13213 (10) dataset contains gene expression data and prognosis information for 117 primary lung adenocarcinoma samples.

The disulfidptosis-related genes (DRGs) were extracted via CRISPR–Cas9 screening from a previous study (8). Genes with |normZ values| > 2 and P values< 0.05, including 399 suppressors and 409 synergists, were further screened to construct a prognostic model.

### 2.2 Prognostic model construction and validation

The chi-square test was used to analyze the differences between the training set, the internal test set and the total dataset in terms of sex, age, tumor stage, depth of invasion (T), lymph node metastasis (N), distal metastasis (M) status and smoking history. The univariate Cox model was used to study the relationship between continuous expression levels of DRGs and OS. The risk ratio (HR) and P value from the univariate Cox regression analysis were used to identify candidate survival-related DRGs. DRGs with an HR > 1 were considered risky DRGs, and those with an HR< 1 were defined as protective DRGs. DRGs that met the criterion of a P value<0.05 were identified as survival-related DRGs and further included in LASSO and multivariate Cox regression analyses to construct a prognostic model. The risk score for each LUAD patient was calculated based on the expression of DRGs (Exp_i_) and Cox coefficients (coef_i_) $\text{Risk score}=\;\sum_{i=1}^{n}Exp_{i}\;\times \;coef_{i}$ . All patients in each dataset were divided into high- or low-risk groups according to the median value. K−M plots were generated to evaluate patient survival in each dataset between the high- and low-risk groups. Moreover, multivariate Cox regression analysis was performed to estimate whether the risk score was independent of clinicopathological features. To investigate the performance of the prognostic model in predicting LUAD patient outcomes, the area under the curve (AUC) of the ROC curve (AUC) was calculated. In addition, the expression of each MRG in the model and its correlation with clinicopathological features were also analyzed.

All analyses were performed with R software (version 4.3.1) and the corresponding fundamental package. The “care” package was used to randomly divide the patients into two datasets at a ratio of 6:4 according to their survival status, which were used as training sets and internal test sets, respectively. The “glmnet” package was used for LASSO regression model analysis. In addition, the “survival” and “survminer” packages were used to perform univariate and multivariate Cox analyses and to generate Kaplan−Meier plots. The “TimeROC” package was used to generate the time-dependent receiver operating characteristic (ROC) curve, and the “survivalROC” package was used to calculate the area under the curve (AUC). Nomogram plots were generated with the “rms” package.

### 2.3 Enrichment analysis

Based on the correlation analysis between the risk score and all mRNAs, gene set enrichment analysis (GSEA) was further performed by using the “ClusterProfiler” package of R software (version 4.3.1).

In addition, the differentially expressed genes (DEGs) between the low and high groups were identified based on the R package “limma” with the thresholds of log(fold change) >1 and P value< 0.05. The DEGs were further input into the DAVID online tool (https://david.ncifcrf.gov/) for pathway and biological process enrichment.

### 2.4 Correlation analysis

To further explore the biological role and clinical significance of the DRG prognostic model, correlation analysis was performed between the risk score and the expression of oncogenes, tumor mutation burden (TMB), immune regulatory gene expression, immune cell infiltration and tumor immune dysfunction and exclusion (TIDE) score. Correlation analysis was performed with the Spearman method based on the “psych” package.

The oncogenes were extracted from the ONGene database (http://www.ongene.bioinfo-minzhao.org) (11). A total of 73 immunomodulatory genes (IMGs) (12) were extracted from previous studies. The immune cell infiltration score was calculated by using the XCELL algorithm (13). Moreover, the TIDE score, dysfunction score and exclusion score of each patient in the datasets were predicted using the TIDE online tool (http://tide.dfci.harvard.edu/) following standard procedures (14).

The Genomics of Drug Sensitivity in Cancer (GDSC) database was developed by the Sanger Research Institute to collect data on the sensitivity and response of tumor cells to drugs (15). “OncoPredict” was used to calculate the drug sensitivity of each sample in the training and validation datasets based on the GDSC V2.0 database (16).

### 2.5 shRNA and overexpression plasmid construction

*CHRNA5* shRNA sequences were designed according to BLOCK-iT™ RNAi Designer (https://rnaidesigner.thermofisher.com/rnaiexpress), and the annealed double-stranded shRNA was cloned and inserted into the pGreen vector. After testing the knockdown efficiency of several candidate shRNAs, the sequence 5’-GGGTCACTATGGAGTTCAAAG-3’ targeting *CHRNA5* and the sequence 5’-GCAGCTGAAATATCCTAAACT-3’ targeting FTO were selected for subsequent experiments. A scrambled nonspecific control shRNA (shNC) was also cloned and inserted into the same vector and used as a negative control. For overexpression, the full-length coding sequence of *CHRNA5* was amplified and cloned and inserted into the pCDH plasmid.

### 2.6 Cell culture and transfection

The human lung cancer cell lines A549 and H1299 were purchased from the American Type Culture Collection (ATCC). All cells were cultured in DMEM (Thermo Fisher Scientific, Inc.) supplemented with 10% FBS (Thermo Fischer Scientific, Inc.) at 37°C in the presence of 5% CO2.

GC cells were seeded in 6-well plates in each well and grown for 24 h. Then, the cells were transfected with 2.5 μg of shCHRNA5 or shNC using Lipofectamine 6000 reagent (Beyotime, China) following the manufacturer’s protocol.

### 2.7 Cell proliferation and migration assays

For cell proliferation, lung cancer cells were initially seeded into 6-well plates. These cells were then incubated with 10 μM EdU for 2 hours. Next, the cells were stabilized with 4% paraformaldehyde and permeabilized using 0.3% Triton X-100, a process conducted in a PBS environment. A subsequent step involved incubating the cells with a click reaction solution, a product provided by the Beyotime Institute of Biotechnology in China. Within a 24-hour timeframe, images of the cells were obtained using an inverted fluorescence microscope, and the resulting data were analyzed with the assistance of NIH ImageJ software (version 1.8.0).

In terms of the cell migration assay, cells from each group were methodically placed in the upper chambers of each Transwell membrane (Corning, Inc., USA). Next, 1 ml of medium without FBS and 2 ml of complete medium were added to the bottom chamber. After a 24-hour incubation period at 37°C in an environment with 5% CO2, the cells were stabilized in methanol and stained with 0.5% crystal violet for 30 minutes. The final stage involved washing the cells in the upper chamber with phosphate-buffered saline (PBS, provided by Gibco, USA) three times. The cells were then imaged using a microscope and evaluated with NIH ImageJ software (version 1.8.0).

### 2.8 Western blot

Total protein from lung cancer cells was extracted using RIPA lysis buffer (Beyotime, China). Protein concentrations were quantified via an Enhanced BCA Kit (Beyotime, China). The proteins, in equivalent quantities, were separated via SDS−PAGE, and 30 μg of each protein was transferred onto a PVDF membrane (Millipore Sigma, Billerica, MA). After blocking with 5% BSA, the membranes were incubated at 4°C overnight with the following primary antibodies: anti-E-cadherin (CDH1, ProteinTech Group, Inc., USA) and anti-N-cadherin (CDH2, ProteinTech Group, Inc., USA), both at a 1:1,000 dilution. Anti-GAPDH (1:1,000 dilution, Cell Signaling Technology Inc., USA) was used as a loading control. The membranes were then incubated with HRP-labeled secondary antibodies for 2 hours at room temperature and subsequently washed three times with TBST. The protein bands were visualized using an enhanced chemiluminescence (ECL) substrate and the GeneTools GBox system (Syngene) and were scanned and quantified with ImageJ software (National Institutes of Health, NIH).

### 2.9 Disulfidptosis assays

Glucose-free DMEM was used to simulate glucose deprivation conditions. When CHRNA5 was knocked down or overexpressed in cells, the culture medium was replaced with glucose-free medium, and the regulatory effect of the gene on dysfildptosis was determined by measuring cell viability and apoptosis.

### 2.10 Statistical analysis

Statistical analyses were conducted using GraphPad V8.3.0 software (GraphPad Software, LLC), and the data are presented as the means ± standard deviations. To ascertain the existence of statistically significant differences between the means of two or more groups, Student’s t test and analysis of variance (ANOVA) were employed. All the statistical tests were two-tailed, and a P value less than 0.05 was considered to indicate statistical significance.

## 3 Manuscript formation

### 3.1 Data collection

Three LUAD cohorts and corresponding clinical data were obtained from the TCGA and GEO databases. The demographic and clinical data for the training, internal testing and independent validation sets are summarized in **Table 1**. After filtering out the samples with missing clinical information from the TCGA LUAD dataset, a total of 504 LUAD patients, including 183 living patients and 321 patients who died at the end of follow-up, were included in this study (median follow-up: 2.474 years). This dataset was randomly divided into a training set (n = 303, 60%) and an internal testing set (n = 201, 40%). As expected, no significant differences were found in the major clinicopathological features between the training, testing and entire TCGA LUAD datasets (**Table 1**). In addition, this study also included a GEO dataset (GSE13213) including 117 LUAD patients, which included 41.88% of deaths at the end of follow-up (median follow-up time was 5.306 years).

**Table 1:** Clinical features of the LUAD patients in the training set, testing set and validation set.

Characteristics	TCGA-LUAD				GSE13213 n = 117
	Training set (60%) n = 303	Testing set (40%) n = 201	All data n = 504	χ2 P value	
Gender					
female	162 (53.47%)	108 (53.73%)	270 (53.57%)	0.998	57 (48.72%)
male	141 (46.53%)	93 (46.27%)	234 (46.43%)		60 (51.28%)
Age					
≤60	95 (31.99%)	63 (31.98%)	158 (31.98%)	1.000	52 (44.44%)
>60	202 (68.01%)	134 (68.02%)	336 (68.02%)		65 (55.56%)
M					
M0	206 (93.64%)	129 (92.14%)	335 (93.06%)	0.863	
M1	14 (6.36%)	11 (7.86%)	25 (6.94%)		
N					
N0	195 (66.33%)	129 (65.82%)	324 (66.12%)	0.993	87 (74.36%)
N1/2/3	99 (33.67%)	67 (34.18%)	166 (33.88%)		30 (25.64%)
T					
T1/2	258 (85.15%)	180 (89.55%)	438 (86.90%)	0.357	104 (88.89%)
T3/4	45 (14.85%)	21 (10.45%)	66 (13.10%)		13 (11.11%)
Stage					
Stage I/II	239 (78.88%)	151 (75.12%)	390 (77.38%)	0.615	79 (67.52%)
Stage III/IV	64 (21.12%)	50 (24.88%)	114 (22.62%)		38 (32.48%)
Smoke history					
Nonsmoke	120 (39.60%)	80 (39.80%)	200 (39.68%)	0.999	
Smoke	183 (60.40%)	121 (60.20%)	304 (60.32%)		
OS time					
≤2	171 (56.44%)	114 (56.72%)	285 (56.55%)	0.998	13 (11.11%)
>2	132 (43.56%)	87 (43.28%)	219 (43.45%)		104 (88.89%)
OS					
Live	188 (62.05%)	133 (66.17%)	321 (63.69%)	0.641	68 (58.12%)
Dead	115 (37.95%)	68 (33.83%)	183 (36.31%)		49 (41.88%)

### 3.2 Construction and validation of the prognostic model according to the DEGs in LUAD patients

**Figure 2:**
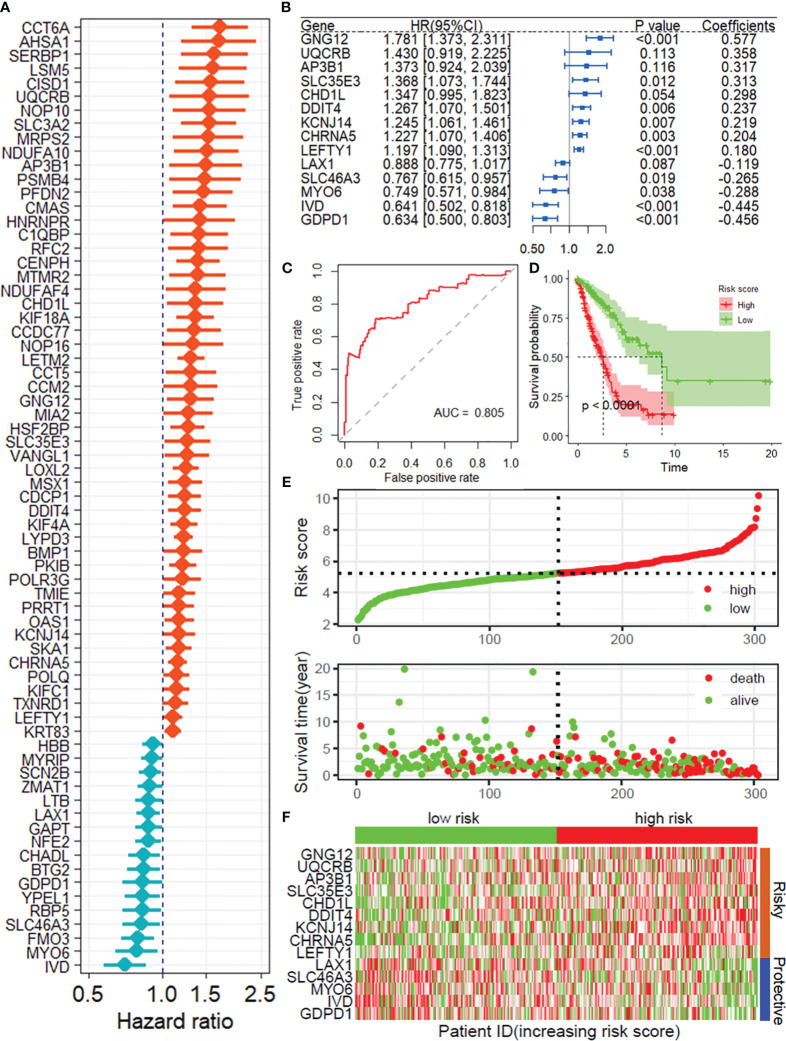
Construction of the prognostic model of DRGs. **(A)** Univariate Cox regression analysis for the selection of DRGs correlated with the OS of LUAD patients. **(B)** Forest plot showing the multivariate Cox regression analysis of 14 DRGs. **(C)** ROC curves for 1-year OS in the training set. **(D)** K−M curve of OS in the training group. **(E)** Risk score distribution and survival status of the training group. **(F)** Heatmap showing the expression of 14 DRGs in the training group. DRGs, disulfidptosis-related genes; OS, Overall survival; ROC, receiver operating characteristic curve.

Based on the CRISPR–Cas9 screenings, a total of 808 DRGs were screened with the criteria of |normZ values| > 2 and P value< 0.05 (**Supplementary Figure 1**). Forty prognosis-related DRGs were identified based on the TCGA training set using univariate Cox regression analysis (**Figure 2A**). Consequently, LASSO-penalized Cox analysis further identified 20 DRGs for multivariate analysis (**Supplementary Figures 2A-B**). The multivariate Cox proportional hazard model was built stepwise using the likelihood-ratio forward method to reach the highest significance. Hence, 14 DRGs were further screened to construct a risk model to assess the prognostic risk of patients with LUAD: risk score = (0.577 × GNG12 Exp) + (0.358 × UQCRB Exp) + (0.317 × AP3B1 Exp) + (0.313 × SLC35E3 Exp) + (0.298 × CHD1L Exp) + (0.237 × DDIT4 Exp) + (0.219 × KCNJ14 Exp) + (0.204 × CHRNA5 Exp) + (0.180 × LEFTY1 Exp) + (-0.119 × LAX1 Exp) + (-0.265 × SLC46A3 Exp) + (-0.288 × MYO6 Exp) + (-0.445 × IVD Exp) + (-0.456 × GDPD1 Exp) (**Figure 2B**). ROC curves demonstrated that the risk score serves as a significant predictor of the OS of LUAD patients, with AUCs greater than 0.730 at 1-5 years (**Figure 2C**). Samples in the training set were classified into low- and high-risk groups based on the median value of the risk score. KM survival analysis indicated that the low-risk group had significantly favorable OS for LUAD patients (**Figure 2D**). The distribution of risk scores between the low-risk and high-risk groups and the survival status and survival time of patients in the two different risk groups are depicted in **Figure 2E**. The relative expression of the 14 DRGs for each patient is shown in **Figure 2F**.

**Figure 3:**
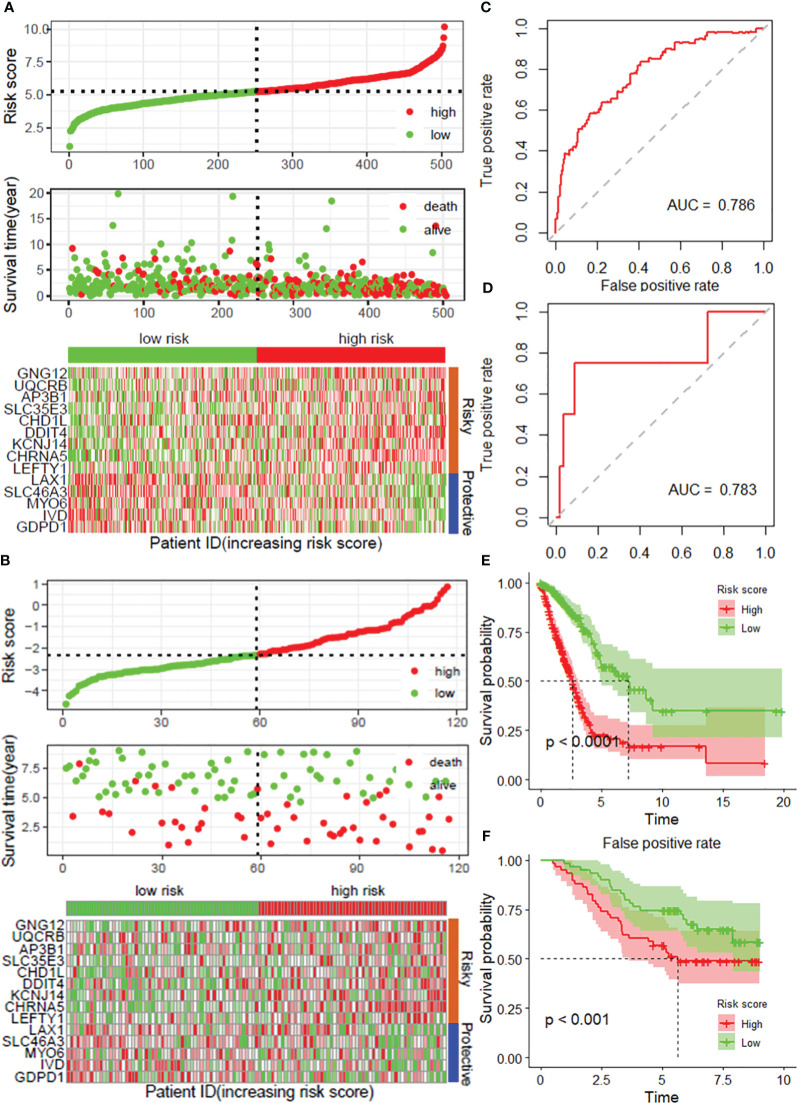
Validation of the prognostic model with 14 DRGs constructed from the training dataset. Risk score distribution, survival status and expression of 14 DRGs in the TCGA-LUAD dataset **(A)** and external validation datasets, *viz*. and GSE31213 **(B)**. ROC curves for overall survival in the TCGA-LUAD **(C)** and GSE31213 **(D)** datasets. K−M curves of OS in the TCGA-LUAD **(E)** and GSE13213 **(F)** datasets. DRGs, disulfidptosis-related genes; ROC, dependent receiver operating curve; TCGA, the cancer genome map; LUAD, lung adenocarcinoma.

To further verify the accuracy and reliability of the prognostic model obtained from the training set, we applied it to the internal testing set and other independent validation cohorts, *viz*. GSE13213. By using the same prognostic model, the classifier could also successfully subdivide patients in the internal testing set (n = 201) into high-risk or low-risk groups with marked differences in overall survival (P = 0.008; **Supplementary Figure 3**). In addition, the same observation was also found in the entire TCGA LUAD dataset (training set and internal testing set, **Figure 3A**), as well as in the GSE13213 validation cohort (**Figure 3B**). Additionally, ROC curves indicated that the risk score was an effective predictor of the OS of LUAD patients in both the TCGA LUAD (**Figure 3C**) and GSE13213 (**Figure 3E**) datasets, with AUCs greater than 0.750. Consistent with the results demonstrated in the training set, the KM survival analysis indicated that the DRG risk score was a significant risk factor for OS in LUAD patients in the above 2 datasets (all *P*< 0.001, **Figure 3D, F**). Importantly, when the other 3 survival indicators, namely, disease-specific survival (DSS), disease-free interval (DFI) and progression-free interval (PFI), were considered, Kaplan–Meier curves and receiver operating characteristic (ROC) curves indicated that the low-risk group had significantly favorable outcomes for LUAD patients (**Supplementary Figure 4**).

### 3.3 The DRG risk score is independent of clinical features

**Figure 4:**
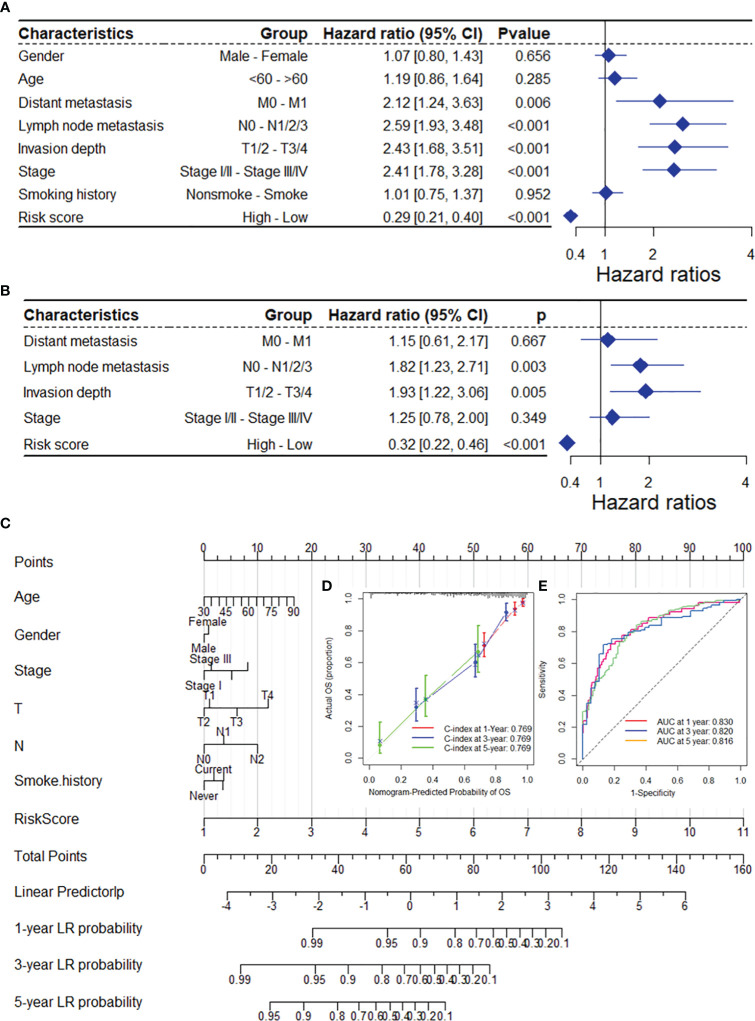
The DRG risk score was an independent prognostic factor for OS in the TCGA-LUAD dataset. Univariate **(A)** and multivariate **(B)** Cox regression analyses of the risk score and clinicopathological features for overall survival in the TCGA-LUAD dataset. **(C)** The nomogram consists of the 14-gene risk score and 6 clinical indicators based on the TCGA-LUAD dataset. The points from these variables are combined, and the locations of the total points are determined. The total points projected on the bottom scales indicate the probabilities of 1-year, 3-year and 5-year overall survival. Calibration plots **(D)** and receiver operating characteristic (ROC) curves **(E)** were used to validate the prognostic nomogram constructed based on the TCGA-LUAD dataset. DRGs, disulfidptosis-related genes; ROC, dependent receiver operating curve; TCGA, the cancer genome map; LUAD, lung adenocarcinoma.

As depicted in **Supplementary Table 1**, the DRG risk score was related to several clinicopathological features in the TCGA-LUAD dataset, including sex, lymph node metastasis, invasion depth and stage. To assess whether the risk score is an independent indicator in LUAD patients, the effect of each clinicopathologic feature on OS was analyzed by univariate Cox regression (**Figure 4A**). As shown in **Figure 4B**, after multivariable adjustment, the risk score remained a powerful and independent factor in the entire TCGA-LUAD dataset. Moreover, the risk score was verified as an independent factor based on the GSE13213 dataset (**Supplementary Figures 5A–B**). The discrepancies in OS stratified by lymph node metastasis (N) and invasion depth (T) stage were analyzed between the low- and high-risk groups in the entire TCGA-LUAD dataset. According to the subgroups classified by N and T stage, the OS of the low-risk group was superior to that of the high-risk group (**Supplementary Figures 6A-D**).

To ensure the robustness and practicability of the 14-DRG prognostic model, a prognostic nomogram for predicting overall survival in LUAD patients was established using the TCGA-LUAD and GSE13213 datasets (**Figure 4C** and **Supplementary Figure 5C**). Major clinicopathological features and risk scores were included in the nomogram. The nomogram was internally validated by computing the bootstrap C-index (≥ 0.700 both in TCGA-LUAD and GSE13213) and a calibration plot (**Figure 4C** and **Supplementary Figure 6E**). The ROC curve confirmed that the score calculated based on the nomogram was highly predictive of overall survival, with AUCs of 0.830 and 0.905 at 1 year in the TCGA-LUAD cohort and GSE13213 cohort, respectively (**Figure 4C** and **Supplementary Figure 6F**).

### 3.4 The DRG risk score is associated with the immune landscape

**Figure 5:**
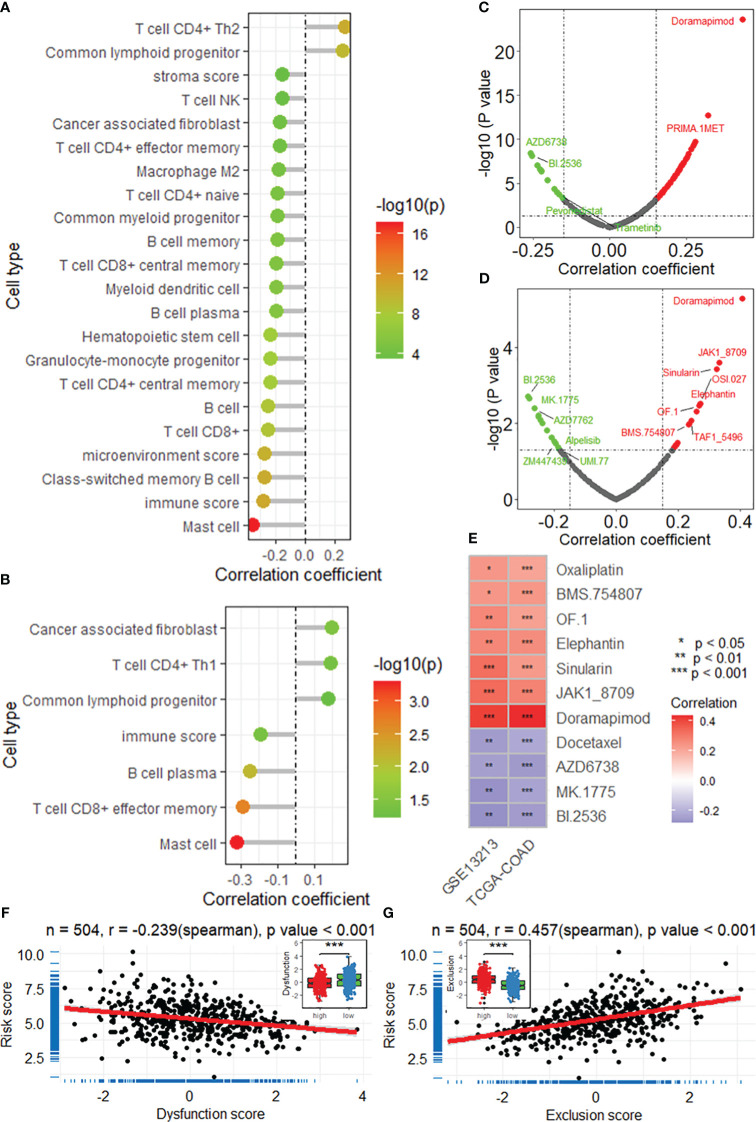
The DRG risk score is correlated with the immune landscape. Lollipop plots showing the results of the correlation analysis between the risk score and immune cell infiltration based on the XCELL algorithm in the TCGA-LUAD **(A)** and GSE13213 **(B)** datasets. Scatter plots showing the results of the correlation analysis between the risk score and sensitivity to antitumor drugs in the TCGA-LUAD **(C)** and GSE13213 **(D)** datasets. **(E)** Heatmap showing the intersection of the drugs significantly correlated with the risk score. *, **, *** represent P value of correlation analysis less than 0.05, 0.01, 0.001, respectively. Correlation analysis between the risk score and dysfunction score **(F)** and exclusion score **(G)**. ***, P value of t-test < 0.001 betweenh igh and low risk groups. DRGs, disulfidptosis-related genes; TCGA, the cancer genome map; LUAD, lung adenocarcinoma.

Based on the XCELL algorithm and TCGA-LUAD dataset, the DRG risk score was found to be associated with infiltration of multiple immune cell types (**Figure 5A**), including CD4+ T cells, Th2 cells, common lymphoid progenitors, mast cells, and B cells, as well as the microenvironment and immune score. Additionally, the risk score was associated with infiltration of many types of immune cells, as was the immune score based on the GSE13213 dataset (**Figure 5B**). In addition, a significant negative correlation between the risk score and dysfunction score was found based on the TIDE algorithm in the TCGA-LUAD dataset (r = -0.239), and the low-risk group had a higher TIDE score (**Figure 5F**). A positive correlation was found between the exclusion score and the risk score (r = 0.457), and the high-risk group had the highest score (**Figure 5G**). After comprehensive consideration of the dysfunction and exclusion scores, a positive correlation was found between the TIDE score and the risk score (r = 0.169), and the high-risk group had a higher score (**Supplementary Figures 7A-B**). Additionally, the same results were found in the GSE13213 validation dataset (**Supplementary Figures 7C-H**). Overall, the TIDE results suggest that the DRG risk score may be associated with poorer immune checkpoint inhibition therapeutic efficacy.

### 3.5 DRG risk score is associated with cancer progression

**Figure 6:**
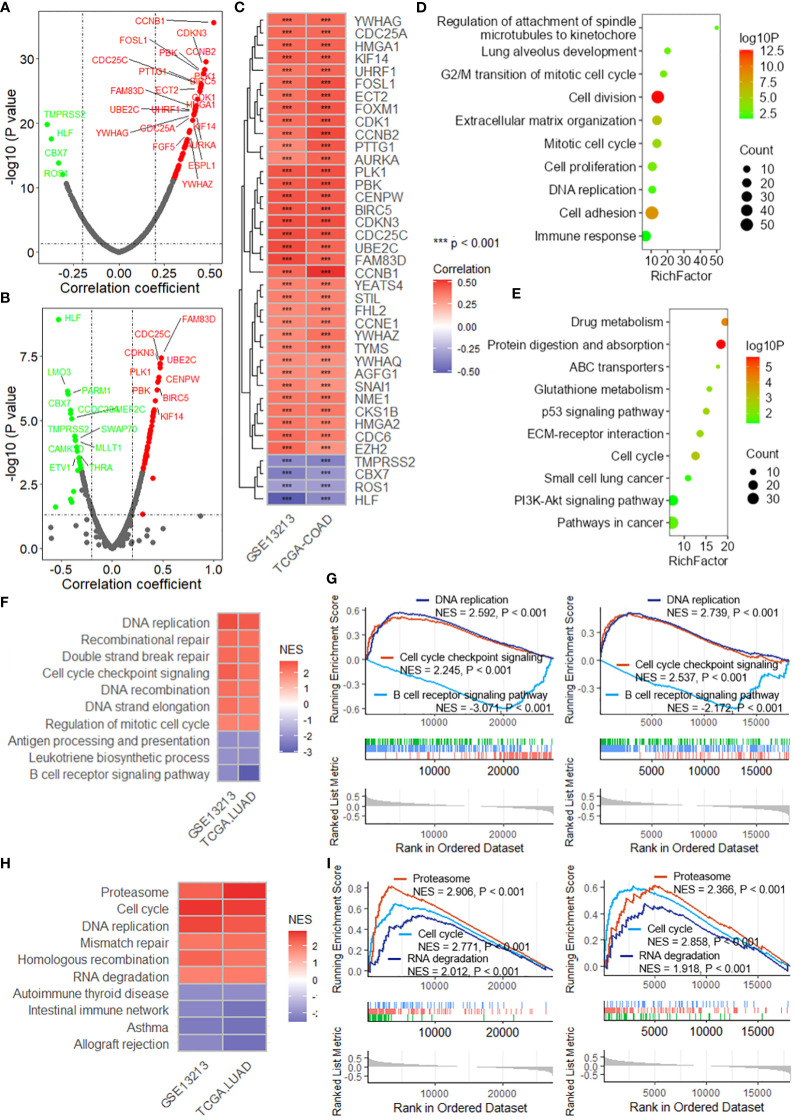
The DRG risk score is associated with cancer progression. Scatter plots showing the results of the correlation analysis between the risk score and oncogenes in the TCGA-LUAD **(A)** and GSE13213 **(B)** datasets. **(C)** Heatmap showing the intersection of the oncogenes significantly correlated with the risk score. *** represent P value of correlation analysis less than 0.001. Lollipop plots showing the enrichment analysis of the differentially expressed genes between the high-risk group and low-risk group for biological processes **(D)** and KEGG pathways **(E)**. **(F)** Heatmap showing the intersecting biological processes in the TCGA-LUAD and GSE13213 datasets based on GSEA. **(G)** GSEA plots showing the enrichment results of three biological processes related to the risk score. **(H)** Heatmap showing the intersecting biological processes in the TCGA-LUAD and GSE13213 datasets based on GSEA. **(I)** GSEA plots showing the enrichment results of three biological processes related to the risk score. GSEA, gene set enrichment analysis; TCGA, the cancer genome map; LUAD, lung adenocarcinoma.

Correlation analysis revealed that the DRG risk score was significantly related to multiple oncogenes in both the TCGA-LUAD (**Figure 6A**) and GSE13213 (**Figure 6B**) datasets. After the intersection of the oncogenes correlated with the risk score in both datasets, 35 positively correlated and 4 negatively correlated oncogenes were identified (**Figure 6C**), including FOSL1, FOXM1, CDK1, and CCNB2. By analyzing the differentially expressed genes (DEGs) between the high-risk and low-risk groups in the TCGA-LUAD and GSE13213 datasets, we obtained a total of 554 genes that were upregulated in both datasets and 401 genes that were downregulated (**Supplementary Figure 8**). The enrichment analysis revealed that these DEGs were significantly enriched in several important biological processes and pathways, including lung alveolus development, G2/M transition of mitotic cell cycle, extracellular matrix organization, cell proliferation, DNA replication, cell adhesion and immune response (**Figure 6D**), as well as drug metabolism, ABC transporters, p53 signaling pathway, ECM-receptor interaction, cell cycle and PI3K-Akt signaling pathway (**Figure 6E**). Moreover, GSEA was performed to investigate the biological processes and pathways potentially related to the DRG risk score. As depicted in **Figure 6F**, the DRG risk score was related to multiple cancer-related biological processes, including DNA replication, recombination repair, double-strand break repair, cell cycle checkpoint signaling and the B-cell receptor signaling pathway, as well as other vital processes, in both the TCGA-LUAD and GSE13213 datasets (**Figure 6G**). Additionally, the risk score was related to several cancer-associated pathways (**Figure 6H**), mainly the proteasome, cell cycle, DNA replication, mismatch repair and RNA degradation pathways, as well as other crucial pathways (**Figure 6I**).

### 3.6 CHRNA5 contributes to lung cancer progression

**Figure 7:**
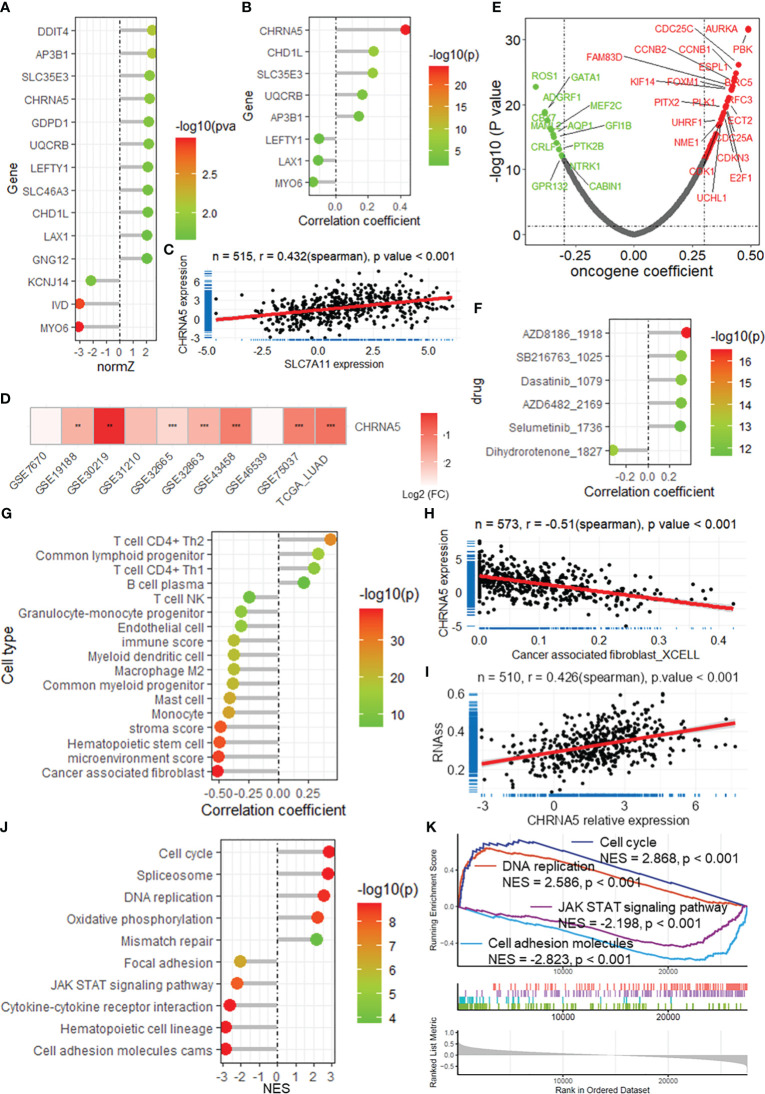
CHRNA5 is highly expressed in LUAD and is related to cancer progression. **(A)** The lollipop plot shows the normalized Z score of the DRGs in the risk model. **(B)** The lollipop plot shows the correlation between SLC7A11 and the DRGs in the risk model. **(C)** Scatter plot showing the correlation between the expression of SLC7A11 and CHRNA5. **(D)** Heatmap showing the change in CHRNA5 expression in tumors compared with normal tissue in multiple datasets. ** and *** represent P value of t-test less than 0.01 and 0.001 between tumor and normal groups, respectively. **(E)** Volcano plot showing the results of the correlation analysis between the expression of CHRNA5 and that of oncogenes. **(F)** Lollipop plot showing the correlation between CHRNA5 and antitumor drug sensitivity. **(G)** Lollipop plot showing the correlation between CHRNA5 and immune cell infiltration. **(H)** Scatter plot showing the correlation between SLC7A11 expression and the infiltration of cancer-associated fibroblasts in the TCGA-LUAD dataset. **(I)** Scatter plot showing the correlation between SLC7A11 expression and tumor stemness calculated by the RNA-seq algorithm based on the TCGA-LUAD dataset. Lollipop plot **(J)** and GSEA plot **(K)** showing the results of GSEA of CHRNA5 for KEGG pathways. GSEA, gene set enrichment analysis; DRGs, disulfidptosis-related genes; TCGA, the cancer genome map; LUAD, lung adenocarcinoma.

Among these DRGs in the constructed risk model, CHRNA5 had a high normalized Z score (normZ = 2.23, **Figure 7A**) and the highest correlation with SLC7A11 (r = 0.432, **Figures 7B-C**). Survival analysis revealed that patients with lower CHRNA5 expression had longer overall survival in both the TCGA-LUAD datasets (**Supplementary Figure 9A**). When considering disease-specific survival and progression-free survival, a better prognosis was found for patients with low CHRNA5 expression (**Supplementary Figures 9B-C**). CHRNA5 expression was greater in tumors than in normal tissues in multiple LUAD datasets (**Figure 7D**). Further correlation analysis revealed that CHRNA5 expression was significantly correlated with multiple oncogenes (**Figure 7E**). Additionally, CHRNA5 expression was positively correlated with the sensitivity to several antitumor drugs (**Figure 7F**). Correlation analysis of immune cell infiltration revealed that CHRNA5 was significantly correlated with several cell types (**Figure 7G**), including DC4+ T cells (Th1/2), cancer-associated fibroblasts (**Figure 7H**), monocytes, mast cells, and M2 macrophages, as well as the microenvironment, stroma and immune score. Moreover, CHRNA5 expression was correlated with tumor stemness in the TCGA-LUAD cohort (**Figure 7I**). GSEA further demonstrated that CHRNA5 is related to many cancer-related KEGG pathways (**Figures 7J-K**) and biological processes (**Supplementary Figure 9D**), including the cell cycle (NES = 2.868), DNA replication (NES = 2.586), the JAK-STAT signaling pathway (NES = -2.198) and cell adhesion molecules (NES = -2.823), as well as several other vital terms.

### 3.7 CHRNA5 regulates proliferation, migration and disulfidptosis in LUAD cells

**Figure 8:**
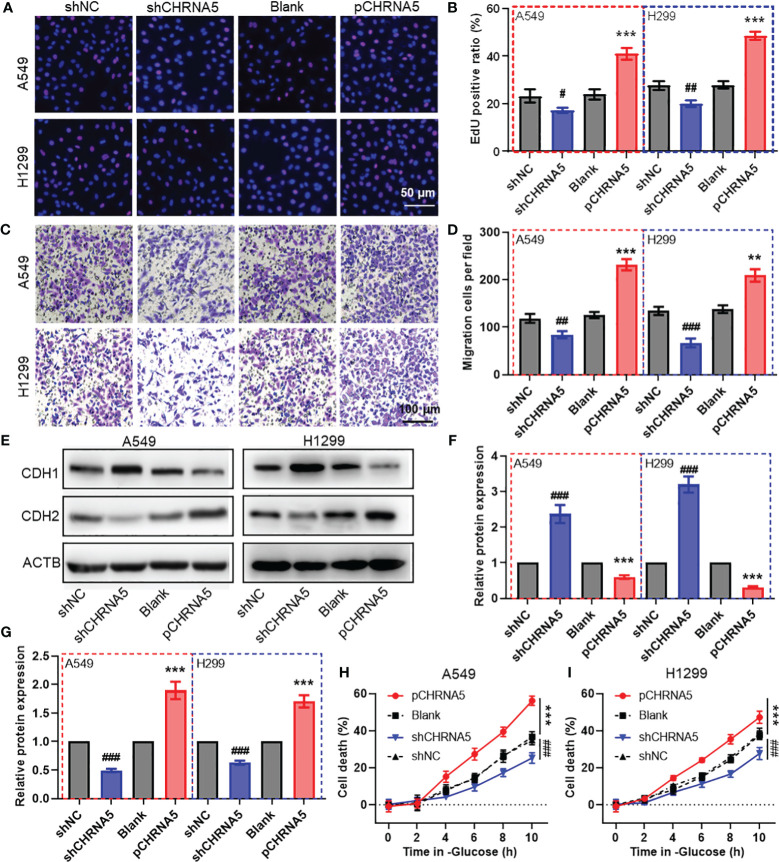
CHRNA5 promotes cell proliferation, migration and disulfidptosis in LUAD. Representative images **(A)** and the quantified results **(B)** of the EdU cell proliferation assay in LUAD cells with CHRNA5 knockdown or knockdown. Representative images **(C)** and the quantified results **(D)** of the transwell cell migration assay in LUAD cells with CHRNA5 knockdown or knockdown. Representative images **(E)** and the quantified results **(F-G)** of western blotting for CDH1 and CDH2 in LUAD cells with CHRNA5 knockdown or expression. A CCK-8 assay was used to measure the cell death induced by glucose deprivation in A549 **(H)** and H1299 **(I)** cells with CHRNA5 knockdown or expression. LUAD, lung adenocarcinoma. #, ## and ### represent P value less than 0.05, 0.01 and 0.001 versus shNC group, respectively. ** and *** represent P value less than 0.05, 0.01 and 0.001 versus Blank group, respectively.

To evaluate the biological function of CHRNA5 in LUAD cells, we constructed shRNA plasmids to knock down CHRNA5 and a plasmid to overexpress CHRNA5 (**Supplementary Figure 10**). EdU assays revealed that CHRNA5 knockdown attenuated LUAD cell proliferation, while CHRNA5 overexpression amplified proliferation in A549 and H1299 cells (**Figures 8A-B**). The transwell migration assay indicated that CHRNA5 knockdown significantly reduced cell migration, while CHRNA5 overexpression significantly increased cell migration (**Figures 8C-D**). The western blotting results demonstrated that CHRNA5 knockdown significantly promoted CDH1 expression (**Figures 8E-F**) but inhibited CDH2 expression (**Figure 8G**). Conversely, CHRNA5 overexpression resulted in the upregulation of CDH2 and the downregulation of CDH1 (**Figures 8E-G**). To further evaluate the synergistic role of CHRNA5 in disulfidptosis, we used glucose-deprived medium to culture LUAD cells. The results of the CCK-8 assay revealed that CHRNA5 knockdown significantly attenuated cell death induced by glucose deprivation, while CHRNA5 overexpression significantly amplified cell death (**Figures 8H-I**).

## 4 Discussion

Disulfidptosis is a new form of regulated cell death in cancers with high SLC7A11 expression under glucose starvation conditions, providing a novel therapeutic strategy for treating malignant tumors (7, 8). Here, we established a prognosis prediction model based on DRGs using LASSO and Cox regression analysis and further screened a key gene in the model, CHRNA5, for functional analysis in lung cancer cells.

Recently, several studies have built risk prediction models for different cancers, including cervical cancer (17), bladder cancer (18, 19), colorectal cancer (20) and lung cancer (21, 22), based on DRGs. With respect to lung cancer, a previous study identified 465 DRGs based on correlation analysis and established a 21-gene-based risk prediction model with an AUC = 0.747 at 1 year (22). Additionally, another study devised a 7-gene-based model with an AUC = 709 at 1 year (21). Compared to these studies, the present study established a risk prediction model with an AUC of 0.805 in lung cancer based on DRGs obtained from CRISPR/Cas9 screening. The superiority of this model was further validated in internal testing and external validation sets, with AUCs of 0.786 and 0.783, respectively. The robustness and practicability of this model were measured by the nomogram, and the nomogram based on the risk score showed better prediction accuracy, with an AUC = 0.830. These results revealed that our DRG risk score model has good predictive accuracy and certain practical value.

The tumor microenvironment (TME) has attracted increasing attention due to its important role in tumor immunosuppression, distant metastasis and drug resistance (23). The TME is mainly composed of tumor cells, infiltrating immune cells, cancer-related stromal cells, endothelial cells and other components (24, 25). The generation and progression of tumors largely depend on external signals received from the surrounding immune cells and nonimmune cells of the TME (26). Our correlation analysis revealed that the risk score was positively correlated with the immune score, microenvironment score and infiltration of mast cells and other cell types. Mast cells are located at the edge of the tumor and TME, usually around blood vessels (27), and have both protumor and antitumor properties. After activation and degranulation, they become highly proinflammatory and actively recruit cells from the innate immune system, mainly neutrophils, macrophages, and eosinophils, as well as cells from the acquired immune system (B cells and T cells), to coordinate antitumor immune responses (28). In contrast, they may also support angiogenesis and MMP9 degradation in the ECM and promote metastasis by releasing VEGF, which is beneficial for tumor progression (28). In addition, the risk score is also correlated with dysfunction and exclusion of T cells, as is the TIDE score, which can predict the clinical response to cancer immunotherapy (14). Further GSEA revealed that the risk score was correlated with DNA replication, the cell cycle, cell adhesion and the immune response, as well as several vital KEGG pathways. Moreover, positive correlations were found between the risk score and the expression of multiple oncogenes and the sensitivity to several antitumor drugs. These results suggest that our DRG risk prediction model may serve as a potential indicator for the prediction of immune microenvironment homeostasis, the evaluation of immune checkpoint blockade therapy, and the evaluation of the biological functional status of tumors.

Among the 14 DRGs included in the risk prediction model, we selected CHRNA5, which had a high normalized Z score based on CRISPR screening and the highest correlation with SLC7A11, to further investigate its regulation of biological function and disulfidptosis in LUAD cells. CHRNA5, a member of the nicotinic acetylcholine receptor superfamily, is a key modulator of nicotine-dependent lung cancer and other malignancies (29, 30). CHRNA5 accelerates lung cancer progression via the MAPK and VEGF pathways (31), influences melanoma growth via Notch1 regulation (32), and promotes radioresistance in oral squamous cell carcinoma by modulating E2F transcription factors (33). In this study, we found that CHRNA5 might function as an oncogene, as evidenced by its upregulation in lung cancer and its positive correlation with oncogene expression. Moreover, survival analysis indicated that patients with high CHRNA5 expression generally have a poorer prognosis. Furthermore, *in vivo* experiments revealed that knocking down CHRNA5 significantly reduced both cell proliferation and migration in LUAD cells. We also investigated its regulatory role in disulfidptosis under glucose-deprived conditions. The results revealed that CHRNA5 knockdown inhibited cell death induced by glucose deprivation, whereas CHRNA5 overexpression enhanced cell death. These findings underscore the significant influence of CHRNA5 on the proliferation and migration of LUAD cells, as well as on disulfidptosis.

In conclusion, our study successfully established and validated a robust risk prediction model rooted in disulfidptosis-related genes (DRGs) for LUAD patients. Notably, this risk score is associated with the homeostasis of the immune microenvironment and the biological function of tumors. CHRNA5, a critical component of this model, has been confirmed to enhance cell proliferation, migration, and disulfidptosis in LUAD cells.

## Data availability statement

The original contributions presented in the study are included in the article/**Supplementary Material**. Further inquiries can be directed to the corresponding author.

## Author contributions

JW: Conceptualization, Funding acquisition, Investigation, Methodology, Project administration, Resources, Software, Writing – original draft. KL: Data curation, Methodology, Software, Validation, Writing – original draft. JWL: Methodology, Writing – review & editing. HZ: Software, Validation, Writing – review & editing. XG: Data curation, Software, Validation, Writing – review & editing. XS: Resources, Writing – review & editing. MW: Software, Writing – review & editing. YH: Resources, Writing – review & editing. JNL: Conceptualization, Project administration, Supervision, Writing – review & editing.
