# Supplementary material for: Identifying and assessing a prognostic model based on disulfidptosis-related genes: implications for immune microenvironment and tumor biology in lung adenocarcinoma
Source: Front Immunol. 2024 May 22;15:1371831. doi: 10.3389/fimmu.2024.1371831 (PMC11150594; doi:10.3389/fimmu.2024.1371831)
Supplement: Supplementary file 1 [file DataSheet_1.docx]

Supplementary Material

# Supplementary Figures and Tables

## Supplementary Table

**Table S1**. DRGs risk score was related to several clinicopathological features in TCGA-LUAD dataset

| Characteristics | | N | mean ± SD | p value |
| --- | --- | --- | --- | --- |
| Gender | female | 270 | 5.147 ± 1.184 | **0.004** |
|  | male | 234 | 5.454 ± 1.169 |  |
| Age | > 60 | 336 | 5.286 ± 1.208 | 0.916 |
|  | ≥ 60 | 158 | 5.298 ± 1.157 |  |
| M | M0 | 335 | 5.323 ± 1.228 | 0.079 |
|  | M1 | 25 | 5.735 ± 1.083 |  |
| N | N0 | 324 | 5.084 ± 1.110 | **< 0.001** |
|  | N1 | 95 | 5.660 ± 1.081 |  |
|  | N2 | 71 | 5.893 ± 1.313 |  |
|  | N3 | 2 | 5.534 ± 0.198 |  |
| T | T1 | 169 | 4.939 ± 0.937 | **< 0.001** |
|  | T2 | 269 | 5.373 ± 1.219 |  |
|  | T3 | 45 | 5.699 ± 1.245 |  |
|  | T4 | 18 | 6.309 ± 1.579 |  |
| Stage | Stage I | 270 | 4.958 ± 1.049 | **< 0.001** |
|  | Stage II | 120 | 5.550 ± 1.112 |  |
|  | Stage III | 80 | 5.965 ± 1.326 |  |
|  | Stage IV | 26 | 5.699 ± 1.077 |  |
| Smoke history | Current | 119 | 5.341 ± 1.160 | 0.342 |
|  | Never | 72 | 5.164 ± 1.113 |  |
|  | Reformed (≤15) | 167 | 5.371 ± 1.226 |  |
|  | Reformed (>15) | 128 | 5.163 ± 1.194 |  |

## Supplementary Figures


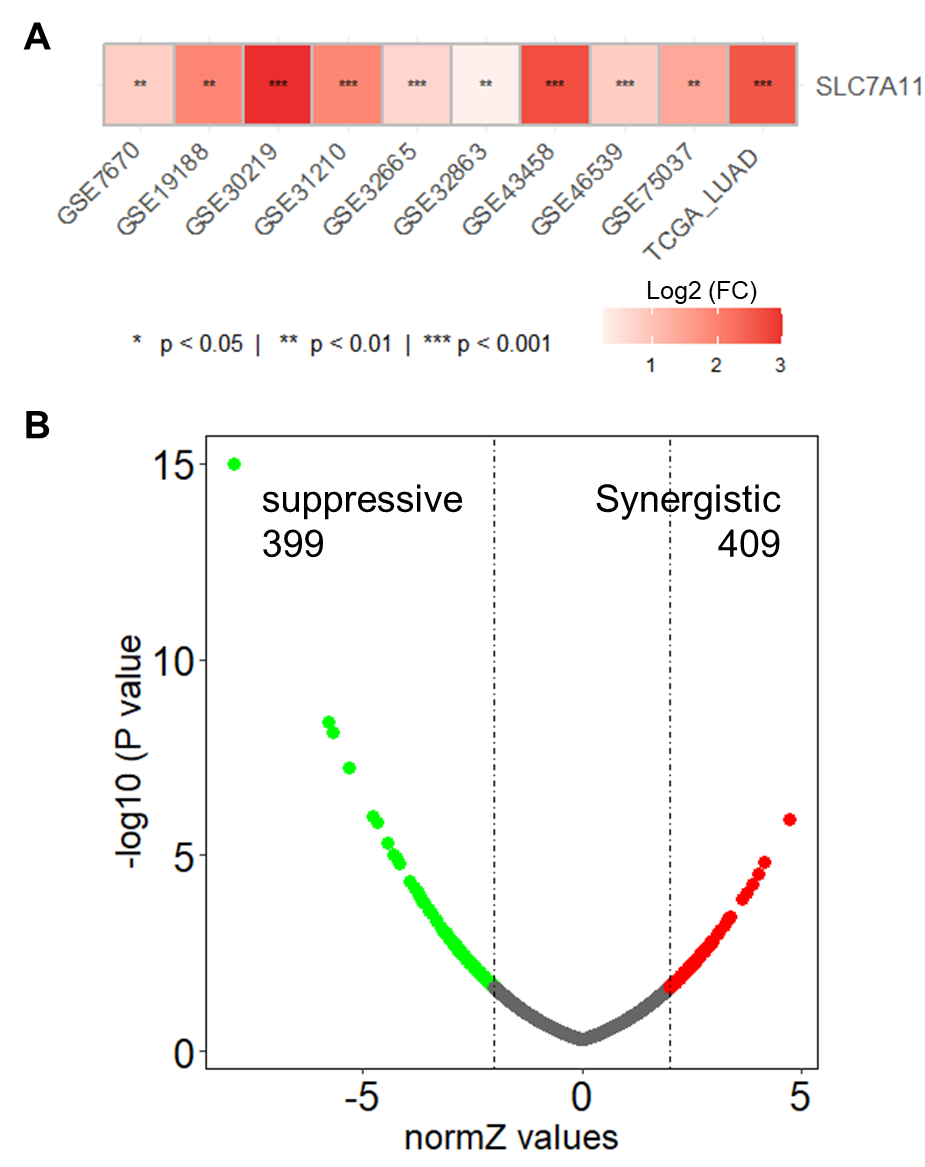


**Figure S1.** Identification of disulfidptosis-related genes. (A) Heatmap shows SLC7A11 was high-expressed in 10 lung cancer datasets. (B) Volcano plot shows the genes with suppressive and synergistic effects in disulfidptosis.


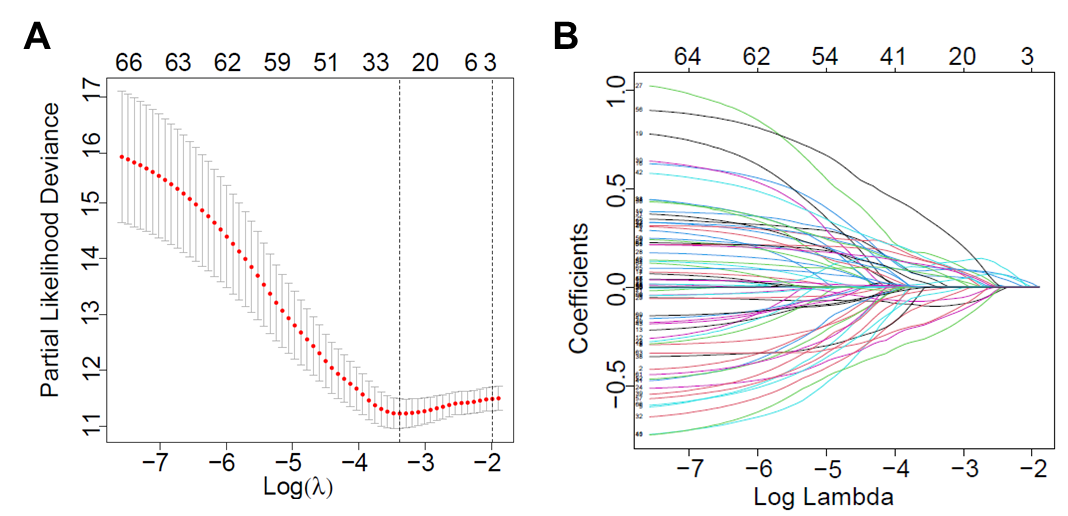


**Figure S2.** LASSO-penalized Cox analysis gives out 23 DRGs for overall survival. (A) Partial likelihood deviance of OS for the LASSO coefficient profiles. (B) LASSO coefficient profiles of the 23 DRGs for OS.


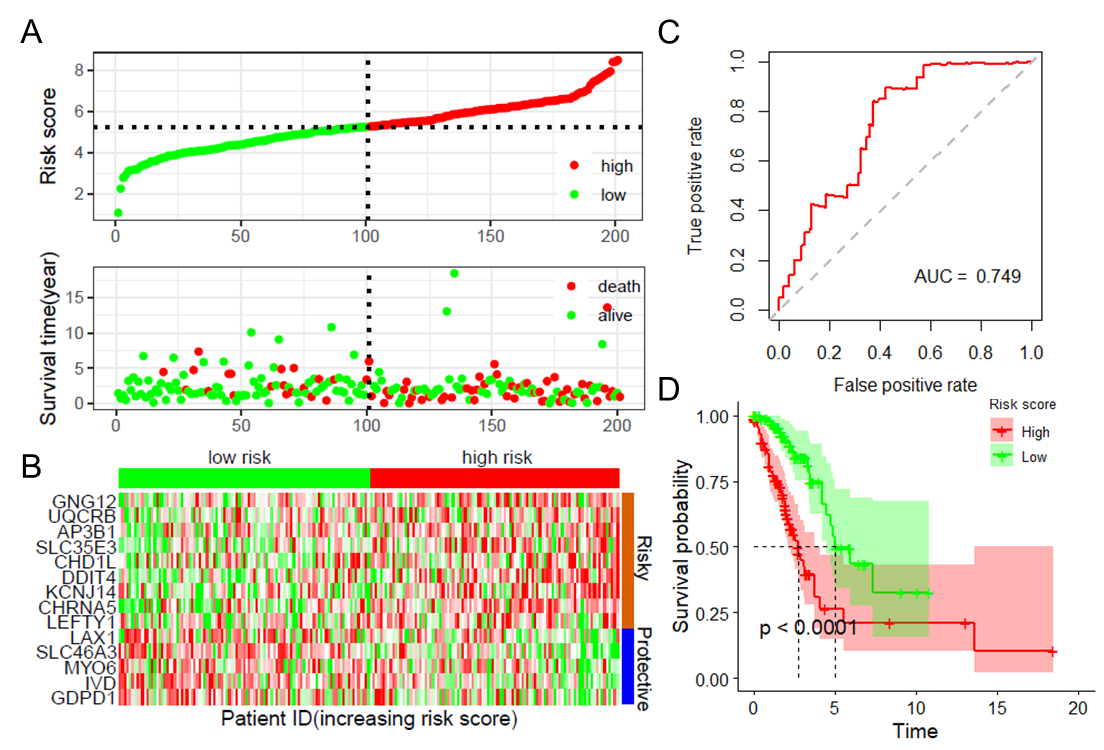


**Figure S3.** Validation of the prognostic model with 14 DRGs constructed by training dataset in internal testing set. Risk score distribution, survival status (A) and the expression of 14 DRGs (B) in internal testing dataset. ROC curve (C) and Kaplan–Meier curve (D) for overall survival in internal testing dataset.


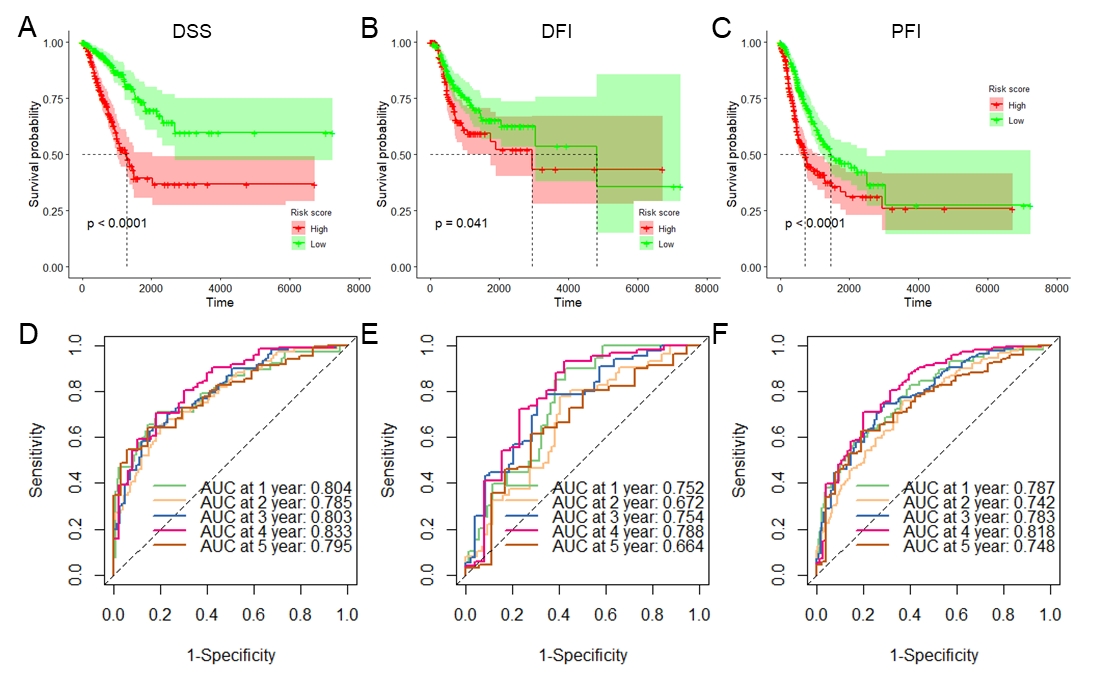


Figure S4. Low-risk group has significantly favorable outcome for the LUAD patients. Kaplan–Meier curve (A-C) and ROC curve (D-F) for DSS, DFI and PFI in TCGA-LUAD dataset. DSS, disease specific survival. DFI, disease free interval. PFI, progression free interval. LUAD, lung adenocarcinoma. ROC, receiver operating characteristic.


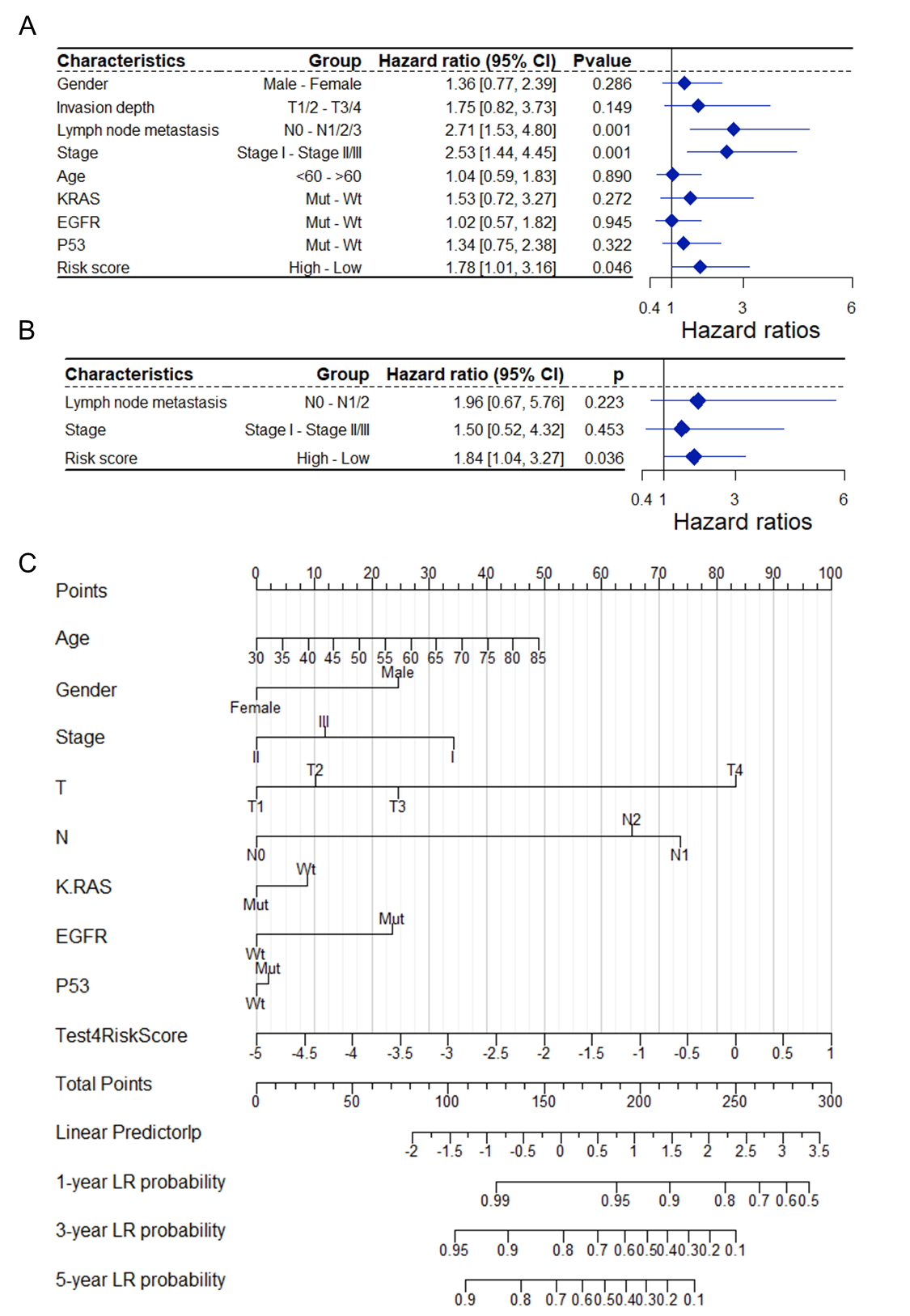


**Figure S5.** DRGs risk score is an independent prognostic factor for overall survival in GSE13213 dataset. Univariate (A) and multivariate (B) Cox regression analyses of risk score and clinic-pathological features for overall survival in GSE13213 dataset. (C) The nomogram consists of the 14-gene risk score and 6 clinical indicators based on GSE13213 dataset. Add the points from these variables together and find the location of the Total Points. The Total Points projected on the bottom scales indicate the probability of 1-year, 3-year and 5-year overall survival. DRGs: disulfidptosis-related genes.


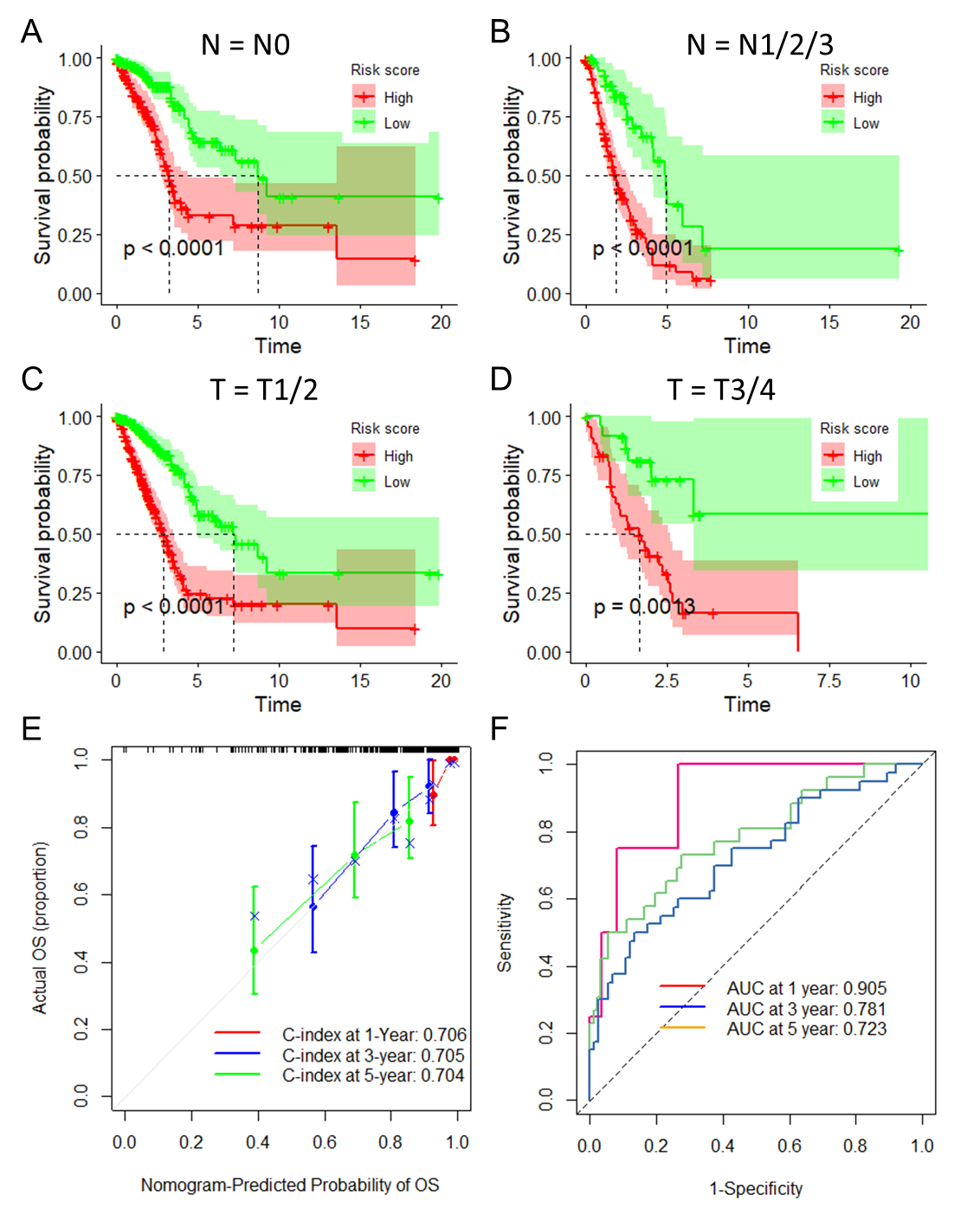


**Figure S6.** DRGs risk score is independent of clinical feature. (A-B) Kaplan-Meier analysis of overall survival stratified by lymph node metastasis. (C-D) Kaplan-Meier analysis of overall survival stratified by invasion depth. Calibration plot (E) and ROC curve (F) were used to validate the prognostic nomogram constructed based on GSE13213 dataset. DRGs: disulfidptosis-related genes. ROC: dependent receiver operating curve.


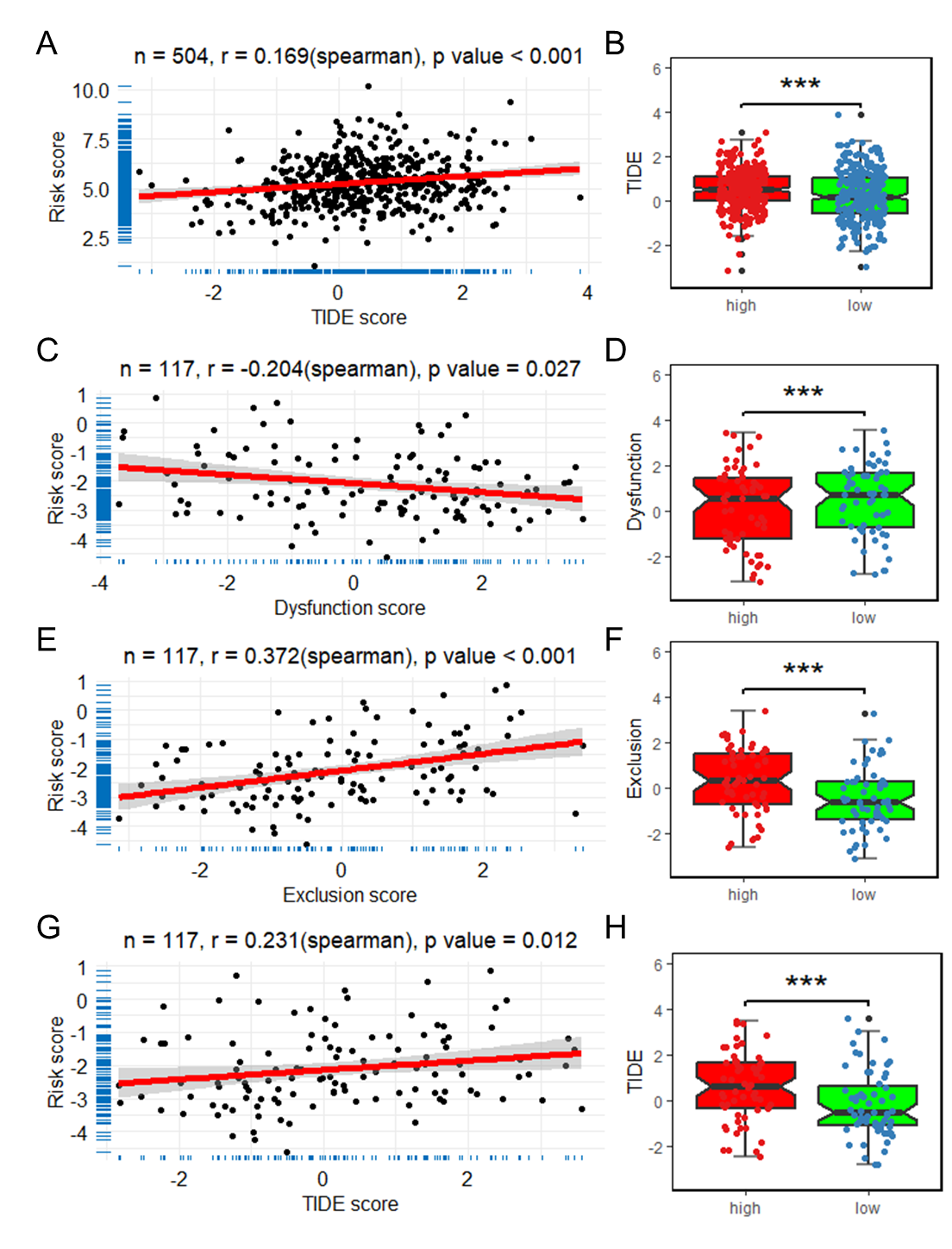


**Figure S7**. The DRGs risk score correlates with TIDE score. (A) Scatter plot shows the correlation between risk score and TIDE score in TCGA-LUAD dataset. (B) Box plot shows the difference of TIDE score between high and low risk groups in TCGA-LUAD dataset. (C) Scatter plot shows the correlation between risk score and dysfunction score in GSE13213 dataset. (D) Box plot shows the difference of dysfunction score between high and low risk groups in GSE13213 dataset. (E) Scatter plot shows the correlation between risk score and exclusion score in GSE13213 dataset. (F) Box plot shows the difference of exclusion score between high and low risk groups in GSE13213 dataset. (G) Scatter plot shows the correlation between risk score and TIDE score in GSE13213 dataset. (H) Box plot shows the difference of TIDE score between high and low risk groups in GSE13213 dataset. DRGs: disulfidptosis-related genes. LUAD: lung adenocacinoma. TIDE, Tumor Immune Dysfunction and Exclusion.


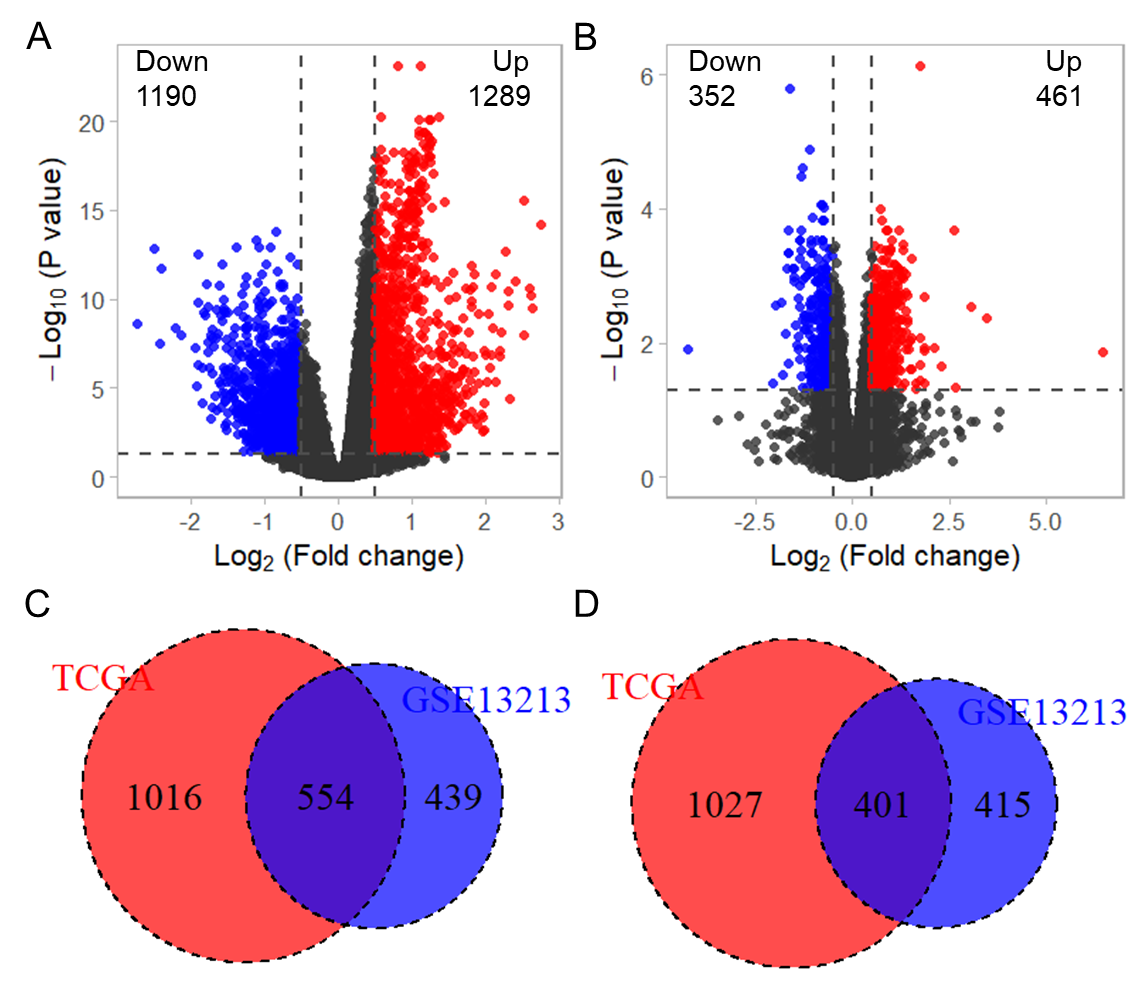


**Figure S8**. Identification of the differently expressed genes between high and low risk groups. Volcano plots show the distribution of the differently expressed genes in TCGA-LUAD (A) and GSE13213 (B) datasets. Venn diagrams show the intersected up-regulated (C) and down-regulated (D) genes in both datasets.


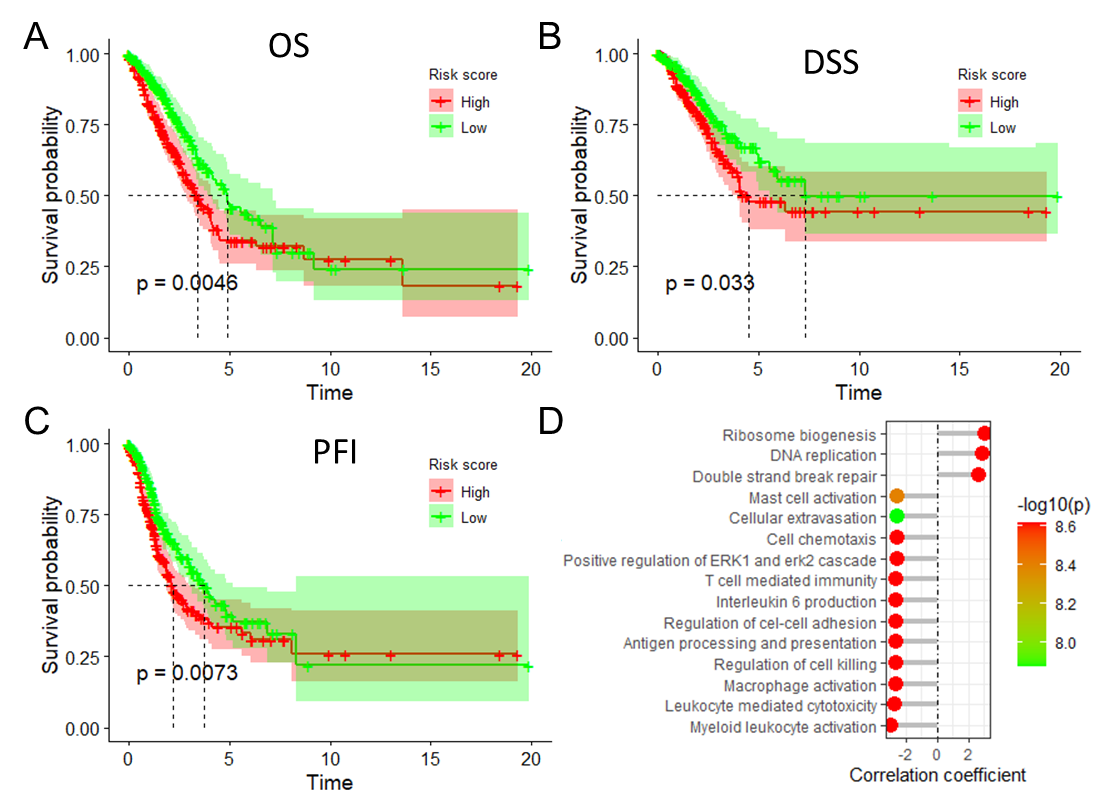


**Figure S9**. CHRNA5 related to lung cancer prognosis. Kaplan–Meier curve of CHRNA5 for OS (A), DSS (B), and PFI (C) in TCGA-LUAD dataset. DSS, disease specific survival. DFI, disease free interval. OS, overall survival. LUAD, lung adenocarcinoma.

**Figure S10**. Validation of CHRNA5 expression in LUAD cells transfected with CHRNA5 overexpressed and knocked down plasmids.
